# Novel Pt-Ag_3_PO_4_/CdS/Chitosan Nanocomposite with Enhanced Photocatalytic and Biological Activities

**DOI:** 10.3390/nano10112320

**Published:** 2020-11-23

**Authors:** Mahsa Kiani, Mojtaba Bagherzadeh, Reyhaneh Kaveh, Navid Rabiee, Yousef Fatahi, Rassoul Dinarvand, Ho Won Jang, Mohammadreza Shokouhimehr, Rajender S. Varma

**Affiliations:** 1 Department of Chemistry, Sharif University of Technology, P.O. Box 11155-3516, Tehran 14155-6451, Iran; mahsa.kiani88@gmail.com (M.K.); reyhanehkaveh@yahoo.com (R.K.); nrabiee94@gmail.com (N.R.); 2 Department of Pharmaceutical Nanotechnology, Faculty of Pharmacy, Tehran University of Medical Sciences, Tehran 14155-6451, Iran; youseffatahi@gmail.com (Y.F.); dinarvand@tums.ac.ir (R.D.); 3 Nanotechnology Research Center, Faculty of Pharmacy, Tehran University of Medical Sciences, Tehran 14155-6451, Iran; 4 Universal Scientific Education and Research Network (USERN), Tehran 15875-4413, Iran; 5 Department of Materials Science and Engineering, Research Institute of Advanced Materials, Seoul National University, Seoul 08826, Korea; hwjang@snu.ac.kr; 6 Regional Center of Advanced Technologies and Materials, Palacky University, Šlechtitelů 27, 78371 Olomouc, Czech Republic

**Keywords:** Pt-Ag_3_PO_4_/CdS/chitosan, photocatalytic activity, antibacterial activity, cytotoxicity, visible light

## Abstract

Decorating photocatalysts with noble metal nanoparticles (e.g., Pt) often increases the catalysts’ photocatalytic activity and biomedical properties. Here, a simple and inexpensive method has been developed to prepare a Pt-Ag_3_PO_4_/CdS/chitosan composite, which was characterized and used for the visible light-induced photocatalytic and antibacterial studies. This synthesized composite showed superior photocatalytic activity for methylene blue degradation as a hazardous pollutant (the maximum dye degradation was observed in 90 min of treatment) and killing of Gram positive bacterial (*Staphylococcus aureus* and *Bacillus cereus*) as well as Gram negative bacteria (*Klebsiella pneumoniae*, *Salmonella typhimurium*, *Escherichia coli*, and *Pseudomonas aeruginosa*) under visible light irradiation. The antibacterial activity of CdS, CdS/Ag_3_PO_4_, and Pt-Ag_3_PO_4_/CdS/chitosan against *E. coli*, *Pseudomonas aeruginosa*, *Salmonella typhimurium*, *Klebsiella pneumoniae*, *Staphylococcus aureus*, and *Bacillus cereus* showed the zone of inhibition (mm) under visible light and under dark conditions at a concentration of 20 µg mL^−1^. Furthermore, the cell viability of the CdS/chitosan, Ag_3_PO_4_, Ag_3_PO_4_/CdS/chitosan, and Pt-Ag_3_PO_4_/CdS/chitosan were investigated on the human embryonic kidney 293 cells (HEK-293), Henrietta Lacks (HeLa), human liver cancer cell line (HepG2), and pheochromocytoma (PC12) cell lines. In addition, the results indicated that the photodegradation rate for Pt-Ag_3_PO_4_/CdS/chitosan is 3.53 times higher than that of CdS and 1.73 times higher than that of the CdS/Ag_3_PO_4_ composite. Moreover, Pt-Ag_3_PO_4_/CdS/chitosan with an optimal amount of CdS killed large areas of different bacteria and different cells separately in a shorter time period under visible-light irradiation, which shows significantly higher efficiency than pure CdS and other CdS/Ag_3_PO_4_ composites. The superb performances of this composite are attributed to its privileged properties, such as retarded recombination of photoinduced electron/hole pairs and a large specific surface area, making Pt-Ag_3_PO_4_/CdS/chitosan a valuable composite that can be deployed for a range of important applications, such as visible light-induced photocatalysis and antibacterial activity.

## 1. Introduction

Scientists have lately focused on antibacterial finishing on different types of textile materials because of their importance in environmental pollutions. In this case, using nanomaterials with improved antibacterial and cellular properties has been raised. The deployment of some transition metal nanoparticles (NPs) offers a practical approach in improving the potential biological properties. Silver is an excellent antibacterial agent because of its chemical and electronic structure, which enables it to penetrate the bacteria’s wall and kill them; the half maximal inhibitory concentration (IC_50_) of silver NPs (AgNPs) is lower than other transition metal NPs, which makes it an appropriate candidate for different biomedical applications [1,2,3,4,5].

Many of the medicinal residues in water, sewage, and pigments in the water line are considered as severe threats to human health [6,7]. Environmental inorganic and organic pollution, created by industries, is a primary concern for human-race and public health because of contaminated foods and waters. Consequently, there is a necessity for industrial-active components and nanomaterials with low cytotoxicity towards different cell lines, for removing the presence of pollutants via dual-functioning nanomaterials [8,9]. There are several potentially toxic and often drug-resistant microorganisms in the environment. For example, species such as *Methicillin-resistant Staphylococcus aureus* (MRSA) are highly prevalent in hospital and municipal wastewater [10]. Several strains of *Escherichia coli*, including problematic strains that are resistant to known drugs, are found in the water [11,12]. Other microorganisms such as *Pseudomonas aeruginosa* and *Salmonella typhimurium* were also found in the river and coastal waters. Thus, immediate steps need to be taken for the development of highly active dual-functional nanomaterials with the potential of working as an antibacterial agent as well as pollutant adsorbents [13].

Pollution from organic contaminants is one of the significant challenges faced by the global society. Estimates indicate that over ten thousand different dyes are used industrially, and over 7 × 10^5^ tons of synthetic dyes are produced all around the world every year. There are a significant number of synthetic dyestuffs in the raw water without any treatment or industrial sewage, which consequently has a considerable impact on the *global environment* [14,15,16,17].

Recently, the field of heterogeneous photocatalysis has expanded rapidly as an efficient way to reduce both the microbial contamination and organic pollution in wastewaters at the same time. The photocatalyst, as the semiconductor material, has two energy bands: the valence band (VB), which is the highest energy band with electrons, and the conduction band (CB), which is the lowest energy band without electrons. In addition, the forbidden energy gap between these two bands is called bandgap energy (E_g_) [18,19,20]. In the photocatalytic process, the exposure of a photocatalyst to light excites it by supplying its band gap energy; consequently, an electron (e^−^) is promoted from the VB to CB, leaving a hole (h^+^) behind. The photoinduced couple (e^−^/h^+^) can reduce or oxidize a contaminant adsorbed on the photocatalyst surface [21,22,23,24]. Furthermore, the nanocomposite can have different application, such as antibacterial and photocatalytic activity. The typical reaction that instigates the antibacterial action is based on the redox reactions on the surface of the material, which lead to the generation of reactive oxygen species (ROS) and react with the cell walls of the bacterial strain [25,26,27].

Silver orthophosphate (Ag_3_PO_4_), a renowned narrow bandgap semiconductor, has attracted intense research interest thanks to its significant photocatalytic activity [28,29,30]. However, the stability of the synthesized nanomaterials depends on the solubility in different solutions, specifically water [31,32,33]. In addition, Ag_3_PO_4_ suffers from photo corrosion of Ag^+^ into Ag^0^ in the process of photocatalytic assay; therefore, composites should be synthesized to limit these detrimental effects [33]. In this manner, two or more different semiconductors could form a novel Ag_3_PO_4_ nanocomposite with an optimized and tunable band energies to facilitate the photocatalytic activities as well as reduce the recombination rate of e^−^/h^+^ pairs. Yao et al. prepared Ag_3_PO_4_/TiO_2_ visible-light composite by depositing Ag_3_PO_4_ NPs onto the surface of TiO_2_ (P-25) [34,35]; this visible light photocatalyst displayed increased activity and is much more stable than pure Ag_3_PO_4_ NPs as it is related to the e^−^/h^+^ efficient separation and the high surface area of the Ag_3_PO_4_/TiO_2_ photocatalyst. At the same time, the increased stability is attributed to the chemical adsorption of Ag^+^ cations in Ag_3_PO_4_ and anions of O^−^ in TiO_2_ [34,36].

Bi et al. have prepared the AgX/Ag_3_PO4 (X = Cl, Br, I) composite via the in situ ion-exchange method, which showed higher photocatalytic activity compared with pure Ag_3_PO_4_ [37,38]. Similarly, Ag_3_PO_4_-based composite photocatalysts including MFe_2_O_4_/Ag_3_PO_4_ (M = Ca, Mg) [32], In(OH)_3_/Ag_3_PO_4_ [39], Ag@(Ag_2_S/Ag_3_PO_4_) [40], and so on have superior photocatalytic performance than single Ag_3_PO_4_.

Cadmium sulfide (CdS) is considered as one of the most promising semiconductors for photocatalytic process, because of its superior ability in absorbing visible light [41,42]. However, these NPs have poor ability in separating the photoinduced e^−^/h^+^ pairs, thus limiting their photocatalytic efficiency. A strategy for decreasing the recombination rate of e^−^/h^+^ pairs on CdS is to combine it with other semiconductors such as NiFe_2_O_4_ [43], ZnO [44], and Ag_3_PO_4_ [45].

Synthesis of multifunctional nanostructured materials has garnered intense attention thanks to their improved physical and chemical properties in electronics, antibacterial, optics, and catalysis. Cadmium sulfide (CdS) is an important II-VI semiconductor with a direct band gap energy of 2.42 eV and has significant applications in dye-sensitized solar cells [46], fluorescence probes [47], displays, photocatalysts [48], and laser light emitting diodes and optoelectronic devices [49] owing to absorption of most of the visible light in the solar spectrum. As for photocatalysts, a high surface area can be the most important factor in certain photocatalytic reactions as they occur on the surface of catalyst in contact with substrates, where reactant molecules must be first adsorbed by the photocatalyst [50].

Kyung Jo et al. prepared the nanocomposite of CdS/Ag_3_PO_4_ and reported its higher visible light photocatalytic activity relative to the pure Ag_3_PO_4_ and CdS nanocrystals, presumably because of the extended life-time of photogenerated e^−^/h^+^ pairs and the enhancement of visible light absorption [51]. Chava et al. fabricated the heteronanostructure of CdS/Ag_3_PO_4_ via solvothermal followed by the chemical reaction method. The photocatalytic hydrogen production rate of this heteronanostructure was evaluated using an aqueous solution containing methanol under visible light illumination; the heteronanostructure exhibited a higher photocatalytic hydrogen evolution rate than that of CdS under visible light irradiation. This showed that the specially formed heterojunctions between CdS and Ag_3_PO_4_ favor the separation of photoinduced electron-hole pairs, which promoted the photocatalytic hydrogen evolution rate [52]. Mirsalari et al. synthesized NPs of CdS and Ag_3_PO_4_ by the sol-gel/mechanochemical method. The increased photocatalytic activity of CdS/Ag_3_PO_4_ nanocomposite towards MB and its lower photoluminescence (PL) intensity can be ascribed to the faster charge carrier’s transfer in the coupled system relative to the individual CdS and Ag_3_PO_4_ precursors [53].

Besides, decorating the prepared photocatalysts with noble metal NPs, such as Pt, is an ideal way to increase the photocatalytic activity and photocatalytic antibacterial properties. In fact, under illumination, the photoinduced electrons in the semiconductor CB can be efficiently directed to the metal NPs attached on the surface, and they can act as sinks for photogenerated electrons. This effect retards the recombination rate of photogenerated electron and hole pairs and prolongs the lifetime of carriers [54].

Chitosan, as an important nature-derived polysaccharide, bearing active functional groups including hydroxyl (–OH) and reactive amino (–NH_2_) groups, has found extensive applications thanks to its low toxicity, significant biocompatibility, adequate water permeability, and high mechanical strength [55,56,57]. Based on the well-established studies, chitosan is commonly used as an adsorbent in environmental science. The functional groups of chitosan could provide plentiful chelation sites for organic pollutants. In the domain of catalysis, chitosan has been selected as a carrier to synthesize composite catalysts for advanced oxidation processes [58,59].

Cao et al. reported the preparation of Ag_3_PO_4_/chitosan/CdS nanocomposite via the in situ growth approach and investigated the photocatalytic activity of this photocatalyst by the decolorization behavior of methyl orange under visible-light illumination [33].

Herein, for the first time, we have synthesized a novel Pt-Ag_3_PO_4_/CdS/chitosan nanocomposite and explored its potential photophysical and biological properties. The presence of Pt NPs in the nanocomposite of Pt-Ag_3_PO_4_/CdS/chitosan can play an important role in the photodegradation process and delays the recombination rate of e^–^/h^+^ pairs. In fact, these NPs with large work functions are used as the electron sinks to trap the photoinduced electrons from the CB of Ag_3_PO_4_, thus enhancing the lifetime of the photoinduced charge carriers. Moreover, these NPs can function as co-catalysts offering the active sites for reduction reactions [60,61,62]. Consequently, the introduction of Pt NPs into the Ag_3_PO_4_/CdS/chitosan nanocomposite has shown significant positive effect on the enhancement of photocatalytic performance.

Interestingly, Prabu et al. prepared the metal colloidal solutions of Pt and Pt-Pd. Antibacterial activity of polyaniline/Pt nanocomposite has been studied with different bacterial strains for food packaging and medical devices, wherein polyaniline has significant antibacterial activity against *E. coli* and *S. aureus* under dark and visible light conditions. Polyaniline, dissolved in dimethyl sulfoxide at different concentrations, displayed antibacterial property against various Gram-positive and Gram-negative bacterial strains and polyaniline incorporated with metal NPs (Pt) also reportedly exhibited enhanced biological activity [63]. The photocatalytic performance of Pt-Ag_3_PO_4_/CdS/chitosan nanocomposite was gauged by photodegradation of methylene blue (MB) as a dangerous pigment under visible light irradiation. Furthermore, extensive antibacterial, antioxidant, and cellular viability studies of this novel 3D nanocomposite were undertaken.

## 2. Experimental

### 2.1. Materials

Chitosan (with the de-acetylation degree of about 85%), extracted from the shells of a shrimp, and K_2_PtCl_2_ were obtained from Sigma-Aldrich, GmbH, Germany. The silver nitrate (AgNO_3_), sodium phosphate monobasic (NaH_2_PO_4_), potassium chloride (KCl), cadmium acetate Cd(CH_3_COO)_2_·2H_2_O, ammonia solution, and MB (C_16_H_18_C_l_N_3_S) were procured from Merck, GmbH, Germany. Other chemicals and solvents such as dimethyl sulfoxide (CH_3_)_2_SO, ethanol (C_2_H_6_O), and distilled water were of analytical grade and were used as received.

### 2.2. Characterization Methods

Room temperature Fourier transform infrared (FTIR) spectra of CdS, chitosan, and Pt-Ag_3_PO_4_/CdS/chitosan nanocomposite were collected with a Bruker Tensor 27 spectrometer (Ettlingen, Germany) using a KBr pellet for sample preparation. The structure of samples was identified by X-ray diffraction (XRD) with an XPERT-PRO diffractometer using Cu *Ka* radiation. Ultraviolet-visible (UV–vis) diffuse reflectance spectra (DRS) were obtained on an Avaspec-2048-TEC UV–vis–near-infrared (NIR) (Avantes, Louisville, CO, USA) spectrometer with barium sulfate (BaSO_4_) powder as the internal standard in order to achieve the optical properties of the samples over a wavelength range of 200–800 nm. All binding energies were determined by this method. An ASAP 2020 surface area analyzer (Micromeritics, GmbH, Germany) was used to carry out the Brunauer-Emmett-Teller (BET) surface area analysis. The morphology of the prepared samples was observed by field emission scanning electron microscope (FESEM, TESCAN MIRA II, TESCAN, Brno, Czech Republic).

The bacteria were spread on nutrient agar plate and cultured at 37 °C for 24 h. A concentration of bacteria equivalent to 10^5^ CFU/mL (colony forming units per milliliter) was prepared by harvesting bacteria in NaCl solution and then the inoculum concentration was determined by McFarland standard and confirmed by serial dilutions.

### 2.3. Synthesis Steps

#### 2.3.1. Synthesis of CdS

For this aim, 2 mmol of Cd(OAc)_2_ was dissolved in 40 mL of dimethyl sulfoxide (DMSO), the mixture was stirred using an ultrasonic bath for 2 h, and the sonicated solution was poured into a 150 mL autoclave (Teflon lined stainless steel) and kept at 130 °C for 28 h. After that, the autoclave was cooled to room temperature and the product (yellow powder) was obtained. The resulting CdS NPs were filtered and washed several times using acetone and ethanol and dried at 60 °C.

#### 2.3.2. Synthesis of CdS/Ag_3_PO_4_ Nanocomposite with a Mass Ratio of 3:7

Briefly, AgNO_3_ (3 mmol) was dissolved in distilled water (40 mL) and a 10 mL aqueous ammonia (1.0 M) was poured into the first solution slowly. Next, CdS (0.38 g) was introduced to the mixture and stirred for about two hours. NaH_2_PO_4_ (0.1 M, 20 mL) was poured into the mixture dropwise and stirred again for one more hour. The obtained precipitates were washed for several times with deionized water and dried at 70 °C. The product CdS/Ag_3_PO_4_ was obtained as a black solid. 

#### 2.3.3. Synthesis of Pt-Ag_3_PO_4_/CdS Nanocomposite

In this step, 0.4 mmol of K_2_PtCl_2_ as well as 9 mmol of KCl were added to 10 mL deionized water in a 100 mL round-bottom flask while stirring constantly. This flask was also heated at 75 °C to completely dissolve the KCl. After cooling the solution to about room temperature, this prepared solution was added to CdS/Ag_3_PO_4_ (450 mg in 25 mL ethanol) slowly. Finally, the obtained suspension was stirred for 4 h and filtered under reduced pressure [64].

#### 2.3.4. Synthesis of Pt-Ag_3_PO_4_/CdS/Chitosan Nanocomposite with a Mass Ratio of 3:2

**Scheme 1:**
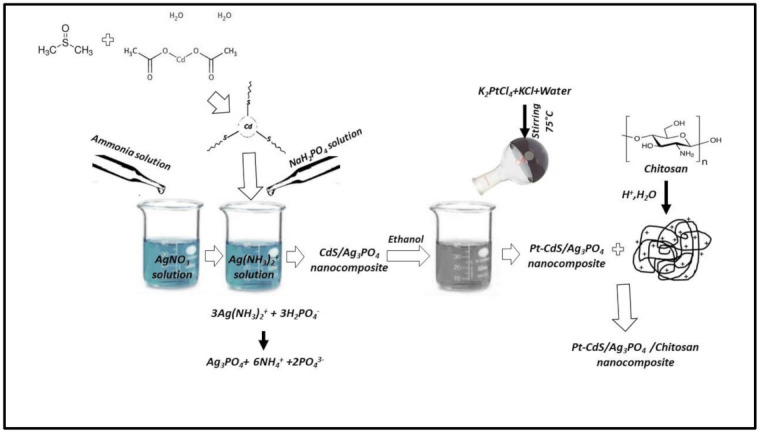
The synthesis process for the Pt-Ag_3_PO_4_/CdS/chitosan nanocomposite.

Briefly, chitosan (0.15 g) was dissolved using acetic acid (1.5%, 30 mL, *v*/*v*); then, an exact amount of Pt-Ag_3_PO_4/_CdS powder (0.225 g) was added to the solution and sonicated for 2 h. The obtained mixture was filtered using a reduced pressure system and washed several times with methanol and deionized water. The product Pt-Ag_3_PO_4_/CdS/chitosan was obtained as a black solid. The synthesis process for the Pt-Ag_3_PO_4_/CdS/chitosan nanocomposite is depicted in Scheme 1.

### 2.4. Photocatalytic Degradation of MB

The photocatalytic ability of the synthesized nanocomposite was evaluated by observation of the MB degradation. For the visible light source, NVSWE21AT was employed, and in all of the measurements, the distance between the light source and MB solution was kept at 3 cm. Figure S1 depicts the emission wavelength of the used NVSWE21AT light source. For each test, 0.01 g of the prepared photocatalyst was added to the 40 mL of MB aqueous solutions with a concentration of 10^−5^ mol L^−1^. Finally, the MB concentration was calculated via monitoring the absorbance spectra at 668 nm. The photocatalytic performance was investigated through the degradation rate (*X*) of MB obtained from the below equation:

*X*(%) = [(*C*_0_*− C_t_*)/*C*_0_] × 100 (1)

where *X*(%) is the degradation rate of MB at time *t* minutes, and *C*_0_ and *C_t_* are MB concentrations at time 0 and *t* minutes, respectively.

After the reaction in each run, the catalyst was collected and dried at 95 °C. Additionally, the experiment for trapping the free radical was conducted to investigate the exact role of reactive species. In this case, *tert*-butyl alcohol (t-BuOH) was selected as the scavenger of hydroxyl radical (•OH), the scavenger of superoxide radical (O_2_^•−^) was chosen to be benzoquinone (BZQ), and the scavenger of hole (h^+^) was selected to be KI; one mM of each scavenger species was added to the mixture in each test.

### 2.5. Antibacterial Test under Visible Light and Dark Conditions

The antibacterial evaluation of Pt-Ag_3_PO_4_/CdS/chitosan nanocomposite, CdS, and CdS/Ag_3_PO_4_ was investigated by the disk-diffusion method. Six bacterial strains named Gram-negative bacteria (*Klebsiella pneumonia*, *Salmonella typhimurium, Pseudomonas aeruginosa,* and *Escherichia coli*) and Gram-positive bacterial (*Bacillus cereus* and *Staphylococcus aureus*) were selected for this assay. The surface of Muller Hinton agar (MHA) was injected via the bacteria by rushing the swab, and afterward, disks were soaked in 0–20 μg/mL of Pt-Ag_3_PO_4_/CdS/chitosan solution that was positioned on the injected agars [65,66]. To ensure antibacterial activity of the final material, two precursors, CdS and CdS/Ag_3_PO_4_, were also tested; they displayed lower antibacterial activity compared with the final material. The standard antibiotic *Penicillin* and *Gentamicin* were used as positive controls and distilled water as negative control in each replicate. The plates were incubated at 37 °C for 2 h under visible light and the dark environment for assessment of the antibacterial activity [67,68]; irradiation was performed under visible light (natural indoor light during the day and 100 W fluorescent lamps overnight) for a certain time.

### 2.6. MTT Assay

Before cell culture, samples were sterilized using ultraviolet exposure followed by washing with ethanol (75%) and phosphate buffer saline (PBS) solution. Cytocompatibility assessment was performed using MTT (3-[4,5-dimethylthiazol-2-yl]-2,5-diphenyltetrazolium bromide) (MTT, Sigma, GmbH, Germany) colorimetric assay at 24 and 48 h. PC12 cells (ATCC CRL-1721^TM^), HEK-293(ATCC CRL-1721^TM^), HeLa (ATCC CCL-2), and HepG2 (ATCC HB 8065) were used for this experiment. Briefly, 1 × 10^5^ cells/well were cultured on the synthesized nanocomposite substrate in Dulbecco’s modified Eagle’s medium (DMEM, Gibco, Thermo Fisher Scientific, GmbH, Germany) containing 100 IU/mL *penicillin*, 100 IU/mL streptomycin (Invitrogen), and 10% fetal bovine serum (FBS; Gibco, Germany) and incubated at 37 °C at 5% CO_2_. At each time point, 100 μL of MTT solution (5 mg/mL in PBS) was added to each well. After 4 h incubation, the medium was removed and the formazone precipitates were dissolved in dimethyl sulfoxide (DMSO; Sigma-Aldrich). The optical absorbance was measured at 570 nm using a microplate Elisa reader (ELX808, BioTek, Bad Friedrichshall, Germany). At least three samples were averaged to calculate each time point.

## 3. Results and Discussion

### 3.1. Characterizations

**Figure 1:**
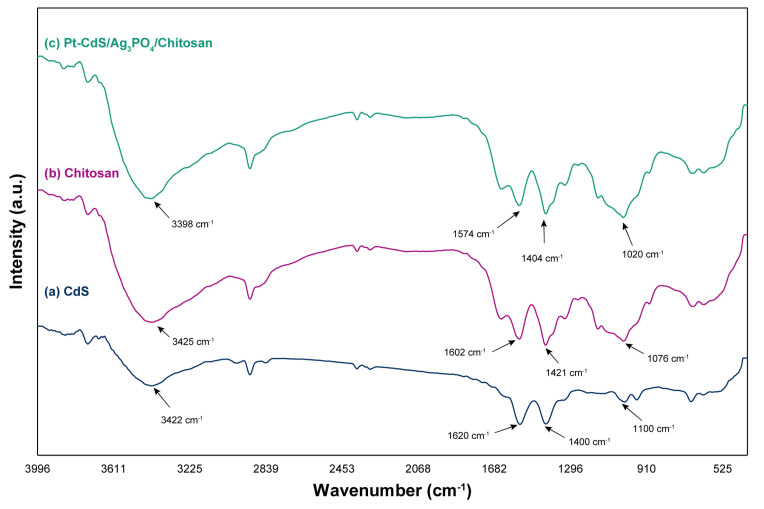
Fourier transform infrared (FTIR) spectra of (**a**) cadmium sulfide (CdS), (**b**) chitosan, and (**c**) Pt-Ag_3_PO_4_/CdS/chitosan (the spectrum of Ag_3_PO_4_ was reproduced with permission from [32]; Copyright John Wiley & Sons, 2018).

The Inductively coupled plasma atomic emission spectroscopy (ICP-AES) result showed that the loading of Pt was 1.19% *w*/*w* Pt. Figure 1 depicts the FTIR spectra of raw CdS, chitosan, and Pt-Ag_3_PO_4_/CdS/chitosan nanocomposites in the range 4000–400 cm^−1^. The FTIR spectrum of CdS showed four characteristic peaks at 3422, 1620, 1400, and 1100 cm^−^^1^, respectively. The peaks at 3422 and 1620 cm^−^^1^ are related to the adsorbed water molecules on the surface of CdS NPs. The FTIR characteristic peaks at 1400 and 1100 cm^−^^1^ are attributed to the Cd-S band [69]. As observed in the FTIR spectrum of Pt-Ag_3_PO_4_/CdS/chitosan nanocomposite, the peak of O-H and N-H stretching vibration at 3425 cm^−1^ in spectrum of chitosan shifted to 3398 cm^−1^, and the peaks of –NH_2_ and –OH at 1602 cm^−1^ and 1421 cm^−1^ moved to 1574 cm^−1^ and 1404 cm^−1^, respectively. These results showed that both the hydroxyl group and the amino group were engaged in the coordination reaction [70]. In addition, the peak of Cd-S bond vibration at 1100 cm^−1^ in the spectrum of CdS became broad and weak and moved to 1020 cm^−1^ in Pt-Ag_3_PO_4_/CdS/chitosan. This result provides more evidence for the fusion of CdS NPs to other NPs in the prepared nanocomposite. Figure S2 shows the FTIR spectrum of Ag_3_PO_4_ NPs, which is based on our previously published study [32]. The band at 3176 cm^−^^1^ is attributed to the O-H stretching of water molecules. The sharp peak at 1388 cm^−^^1^ is related to the antisymmetric stretching mode of the PO_4_^3^^−^ group, whereas the corresponding symmetric vibration mode is found at 763 cm^−^^1^. The asymmetric stretching vibration of P-O bonds is found at 1020 cm^−^^1^, whereas the peak at 547 cm^−^^1^ is attributed to the asymmetric bending mode of P-O bonds [71].

**Figure 2:**
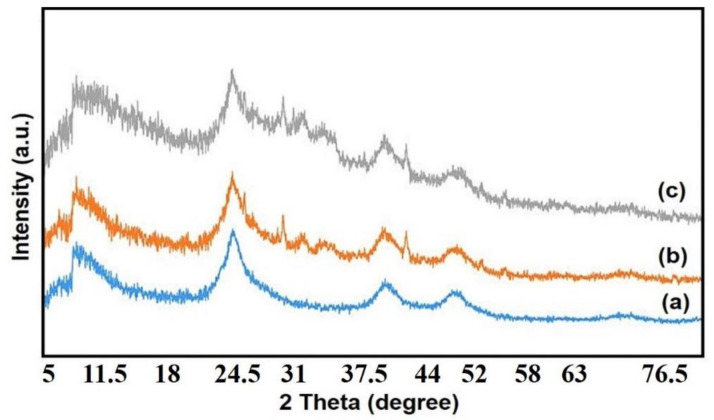
X-ray diffraction (XRD) patterns of (**a**) CdS, (**b**) Pt-Ag_3_PO_4_/CdS/chitosan, and (**c**) CdS/Ag_3_PO_4_.

XRD patterns for CdS, CdS/Ag_3_PO_4_, and Pt-Ag_3_PO_4_/CdS/chitosan nanocomposite are depicted in Figure 2. The XRD pattern of pure CdS showed characterization peaks at 26.6°, 44°, and 51.9°, which were related to the (111), (220), and (311) planes, respectively (JCPDS 89-0440). In the XRD pattern of Pt-Ag_3_PO_4_/CdS/chitosan in Figure 2, the diffraction peak at ~27 of CdS and the peaks at 32.2°, 33.9°, 36.9°, 43.5°, 46°, 54.8°, and 57°, assigned to the (200), (210), (211), (220), (310), (320), and (321) planes of Ag_3_PO_4_, respectively, were vividly observed. Because of the low Pt loading on CdS/Ag_3_PO_4_/chitosan nanocomposite, no Pt diffraction peaks were detected [43,72]. XRD patterns for Ag_3_PO_4_ are shown in Figure S3. All diffraction peaks can be indexed to Ag_3_PO_4_ (JCPDS NO. 6-0505).

**Figure 3:**
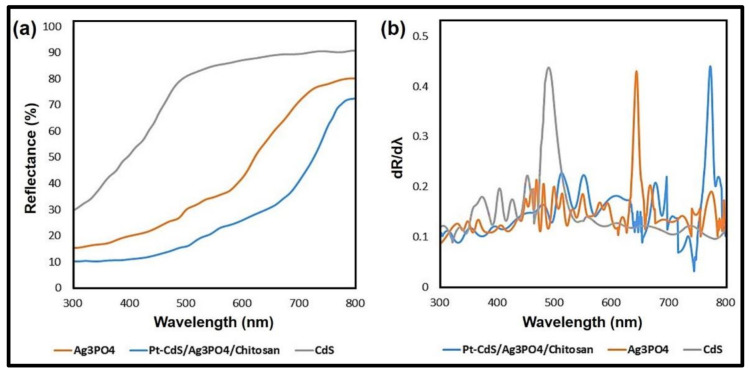
(**a**) The UV–vis diffuse reflectance spectra (DRS) of CdS, Ag_3_PO_4_, and Pt-Ag_3_PO_4_/CdS/chitosan; (**b**) the corresponding first derivative spectra.

The optical properties of the synthesized samples were studied by the diffuse reflectance spectra (DRS). Figure 3a depicts the DRS of CdS, Ag_3_PO_4_, and Pt-Ag_3_PO_4_/CdS/chitosan samples with clear red shift and intense absorptions in the wavelength ranging from 400 to 800 nm. Figure 3b shows the corresponding first derivative of the aforementioned spectra. The absorption edge of the synthesized samples can be obtained from the peaks on the first derivative spectra. Based on the attained results, CdS and Ag_3_PO_4_ have an absorption edge at 491 and 644 nm, respectively. According to the absorption edge, the band gap energy (Eg) of CdS and Ag_3_PO_4_ is calculated to be 2.52 and 1.92 eV, respectively. It should be mentioned that the Eg of these two samples are slightly different from standard values, which may be related to the different preparative conditions of the process [32,73]. The absorption edge of Pt-Ag_3_PO_4_/CdS/chitosan is located at 773 nm, implying a band gap energy of 1.60 eV. Therefore, the Pt-Ag_3_PO_4_/CdS/chitosan nanocomposite could have a good photocatalytic performance under visible light irradiation [74,75,76,77,78].

**Figure 4:**
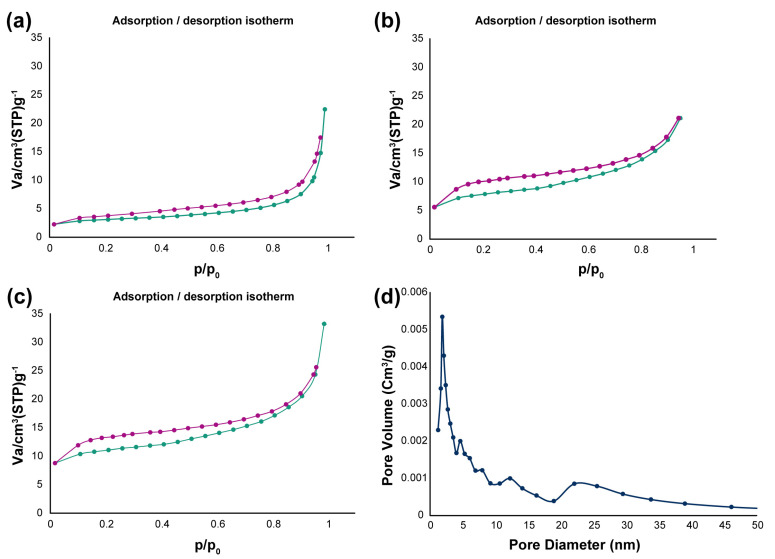
BET surface area of (**a**) CdS, (**b**) CdS/Ag_3_PO_4_, and (**c**) Pt-Ag_3_PO_4_/CdS/chitosan. (**d**) Pore size distribution plot (BJH plot) of Pt-Ag_3_PO_4_/CdS/chitosan.

The surface area of the prepared samples was investigated by BET analysis (Figure 4a–c). According to the BET results, the surface area values were found to be 10.796 m^2^/g, 21 m^2^/g, and 40.809 m^2^/g for CdS, CdS/Ag_3_PO_4_, and Pt-Ag_3_PO_4_/CdS/chitosan, respectively. The higher BET specific surface area of the CdS/Ag_3_PO_4_ composite compared with pure CdS can be ascribed to the introduction of Ag_3_PO_4_ particles in the composite and is beneficial to improve the catalytic performance. Furthermore, the nanocomposite of Pt-Ag_3_PO_4_/CdS/chitosan has a higher specific surface area compared with CdS/Ag_3_PO_4_, which is mainly due to the presence of chitosan. In fact, the matrix of chitosan effectively prevented the aggregation of the NPs, as highly dispersed NPs on chitosan matrix are obtained. Finally, the high specific surface area of the Pt-Ag_3_PO_4_/CdS/chitosan nanocomposite showed significant adsorption capacity of this sample, and it is one of the main reasons for its high photocatalytic activity. The Barrett, Joyner, Halenda (BJH) plot for Pt-Ag_3_PO_4_/CdS/chitosan nanocomposite, in Figure 4d, depicts that the mesopore size distribution estimated by the BJH method is around 1.85 nm.

**Figure 5:**
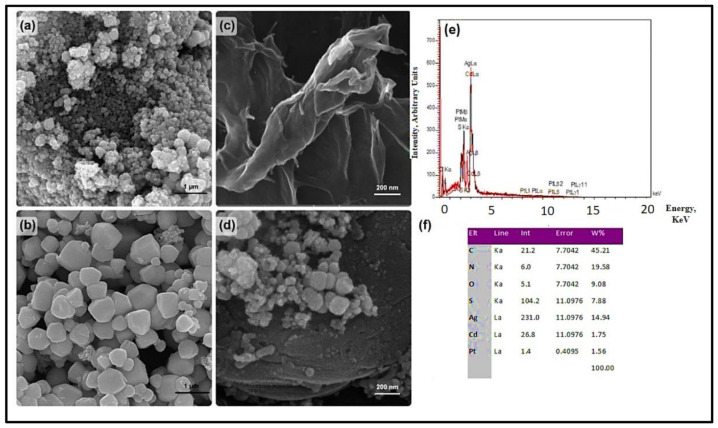
Field emission scanning electron microscope (FESEM) image of (**a**) CdS, (**b**) Ag_3_PO_4_, (**c**) chitosan, and (**d**) Pt-Ag_3_PO_4_/CdS/chitosan. (**e**) EDS spectrum of Pt-Ag_3_PO_4_/CdS/chitosan. (**f**) EDS table of Pt-Ag_3_PO_4_/CdS/chitosan.

**Figure 6:**
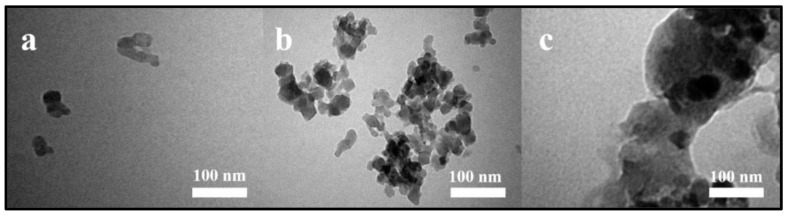
TEM image of (**a**) CdS, (**b**) Ag_3_PO_4_, and (**c**) Pt-Ag_3_PO_4_/CdS/chitosan.

FESEM micrograph was utilized to investigate the morphology of the CdS, Ag_3_PO_4_, chitosan, and Pt-Ag_3_PO_4_/CdS/chitosan samples. Spherical CdS NPs with several aggregations were observed in Figure 5a, whereas Figure 5b shows the morphology of the prepared Ag_3_PO_4_ NPs, which have irregular particles. Figure 5c depicts that chitosan has a flaky nature with a relatively rough surface, which can be related to strong interactions between chitosan molecules. The FESEM image of Pt-Ag_3_PO_4_/CdS/chitosan in Figure 5d exhibits the presence of spherical CdS NPs as well as irregular particles of Ag_3_PO_4_ in the nanocomposite of Pt-Ag_3_PO_4_/CdS/chitosan. The presence of NPs on the surface of chitosan as the matrix is clearly observed in Figure 5d. Energy dispersive X-ray (EDX) analysis (Figure 5e) confirms the composition of the NPs, which illustrated that Pt-Ag_3_PO_4_/CdS/chitosan comprises Pt, Cd, S, Ag, O, and C elements. All of these results are in good agreement with the transmission electron microscopy (TEM) images as shown in Figure 6 and the EDX table in Figure 5f. Based on the TEM images, the size of the nanocomposites is in the range of 80 to 150 nm, which is in good agreement with the FESEM images. In addition, some aggregations were observed compared with the CdS and Ag_3_PO_4_ compartments, which are due to the presence of chitosan substrate as well as different NPs.

### 3.2. Photocatalytic Performance for MB Degradation

**Figure 7:**
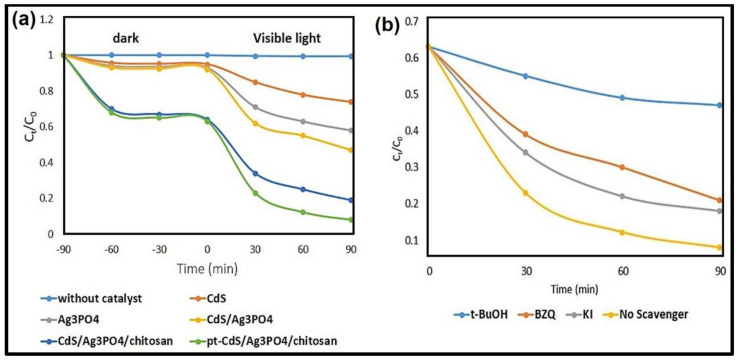
(**a**) The degradation efficiency for methylene blue (MB) (10^−5^ mol/L) removal in the process of prepared samples under visible light irradiation. (**b**) Effect of various scavengers on the degradation of MB by Pt-Ag_3_PO_4_/CdS/chitosan.

The maximum photodegradation rate of MB was obtained through the use of Pt-Ag_3_PO_4_/CdS/chitosan catalyst. As shown in Figure 7a, MB was stable in the dark situation or under visible-light irradiation in the absence of any catalysts. NPs of CdS, Ag_3_PO_4_, and CdS/Ag_3_PO_4_ adsorbed a little amount of MB under dark conditions. However, Pt-Ag_3_PO_4_/CdS/chitosan composite showed perfect adsorption capacity (~37% in dark time) and the dye removal rate increased (~92% at 90 min) for MB. The experimental results revealed that the percentage of MB removal was found to be 53% and 81% in the presence of the CdS/Ag_3_PO_4_ and CdS/Ag_3_PO_4_/chitosan photocatalyst, respectively.

The improved photocatalytic performance of the CdS/Ag_3_PO_4_ sample compared with pure CdS is attributed to the presence of Ag_3_PO_4_ NPs. The photogenerated electrons were transferred from the CdS CB into the Ag_3_PO_4_ CB and the holes that had been photogenerated were transferred from the VB of Ag_3_PO_4_ into the VB of CdS. This can reduce the recombination rate of e^−^/h^+^ pairs.

Moreover, the introduction of chitosan as a good carrier into CdS/Ag_3_PO_4_ nanocomposite increased the adsorption of dye, and this issue can be effective in improving the performance of the prepared nanocomposite.

In addition, because of the presence of chitosan matrix and Pt NPs in the CdS/Ag_3_PO_4_/chitosan nanocomposite, the catalytic performance of this photocatalyst significantly increased; Pt NPs can play an important role in the photodegradation process as they delay the recombination rate of e^–^/h^+^ pairs. In fact, these NPs with large work functions are used as the electron sinks to trap the photoinduced electrons from the CB of Ag_3_PO_4_, thus the lifetime of the photoinduced charge carriers can be prolonged.

### 3.3. Possible Photocatalytic Mechanism

In order to increase the photocatalytic activity of a catalyst, it is imperative to use various structures with matched bands of different semiconductors for the formation and the separation of photoinduced e^−^/h^+^ pairs. The bandgap for CdS and Ag_3_PO_4_ is 2.52 eV and 1.92 eV, respectively [32,43]. The CB potential of CdS is more negative than that CB of Ag_3_PO_4_. The photoinduced electrons on the CB of CdS migrate to the CB of Ag_3_PO_4_ and the VB photogenerated holes of Ag_3_PO_4_ could migrate to CdS. The CdS and Ag_3_PO_4_ electron transfer was assessed by the energy of bandgap of Pt-Ag_3_PO_4_/CdS/chitosan composite (1.6 eV). It should be noted that Ag_3_PO_4_ and CdS NPs are intimately connected by the matrix of the chitosan in Pt-Ag_3_PO_4_/CdS/chitosan composite. 

The ability of adsorption and formation of e^−^/h^+^ pairs have a direct relationship with the photocatalytic efficiency of the synthesized nanocomposite; Pt NPs play the role of a co-catalyst in the photodegradation process and delay the recombination rate of e^–^/h^+^ pairs. 

According to the BET results (Figure 4c), the Pt-Ag_3_PO_4_/CdS/chitosan composite possessed a large specific surface area (40.809 m^2^/g). In fact, Pt-Ag_3_PO_4/_CdS photocatalyst onto the chitosan matrix effectively prevented the aggregation of the NPs and increased the specific surface area of the prepared catalyst. 

The proposed photocatalytic reaction mechanism of Pt-Ag_3_PO_4_/CdS/chitosan composite can be expressed as follows.

**Figure 8:**
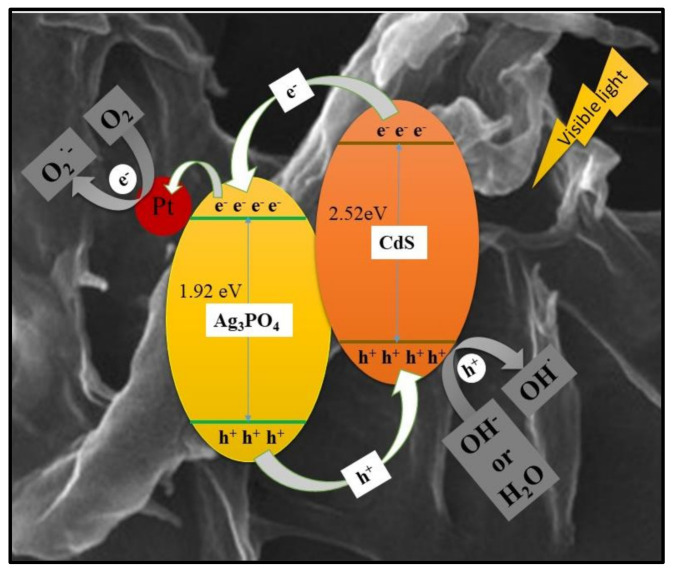
Proposed photocatalytic mechanism of Pt-Ag_3_PO_4_/CdS/chitosan under visible light.

Initially, both CdS and Ag_3_PO_4_ were excited simultaneously to form a pair of e^−^/h^+^ driven by irradiation (visible light), after which the electrons that had been photogenerated were transferred from the CdS CB into the Ag_3_PO_4_ CB, and then electrons transport from the Ag_3_PO_4_ CB to the Pt NPs. In this step, the Pt NPs efficiently trap the photogenerated electrons because of their capacity in the reservoir of the e^−^. On the other hand, the holes that had been photogenerated were transferred from the VB of Ag_3_PO_4_ into the VB of CdS. Finally, The photogenerated electrons react with O_2_ to generate O_2_^•−^, which then reacts with H^+^ to form •OOH, with the quick decomposition to •OH. Additionally, the holes oxidize H_2_O and –OH to form •OH, an important species in the process of photodegradation of organic pollutants. So, the process of •OH radicals’ formation can occur through two pathways, as shown in Figure 8. In conclusion, the prepared catalyst of Pt-Ag_3_PO_4_/CdS/chitosan efficiently decreases the recombination rate of e^−^/h^+^ charge carriers, thus enhancing the photocatalytic effectively and avoiding the photo corrosion of Ag_3_PO_4_ (Ag^+^ + e^−^ → Ag). Thus, the Pt-Ag_3_PO_4_/CdS/chitosan catalyst is significantly stable and active in comparison with pure CdS or Ag_3_PO_4_ [33].

### 3.4. Effect of Reactive Species on Photodegradation of MB

To investigate the possible photodegradation mechanism of MB by Pt-Ag_3_PO_4_/CdS/chitosan, various scavengers were added to the mixture in order to quench the relevant active species. In this study, *t*-BuOH, BZQ, and KI were chosen to be the scavengers of hydroxyl radicals (•OH), superoxide radicals (O_2_^•−^), and holes (h^+^), respectively. As depicted in Figure 7b, the photocatalytic degradation efficiency for MB with Pt-Ag_3_PO_4_/CdS/chitosan was about 92% after 90 min under visible light illumination, which was affected slightly in the presence of BZQ and KI, demonstrating that superoxide radicals and holes played a small role in the photodegradation of MB. However, the photocatalytic activity of the Pt-Ag_3_PO_4_/CdS/chitosan was completely suppressed by *t*-BuOH, indicating that •OH plays the most important role in this photodegradation process; the percentage of MB removal was about 53%, 79%, and 82% in the presence of *t*-BuOH, BZQ, and KI scavengers, respectively. Consequently, the photodegradation of MB was lessened most remarkably in the presence of *t*-BuOH, suggesting that oxidation reaction was mediated mainly via hydroxyl radicals.

### 3.5. Recyclability of Pt-Ag_3_PO_4_/CdS/Chitosan Nanocomposites

**Figure 9:**
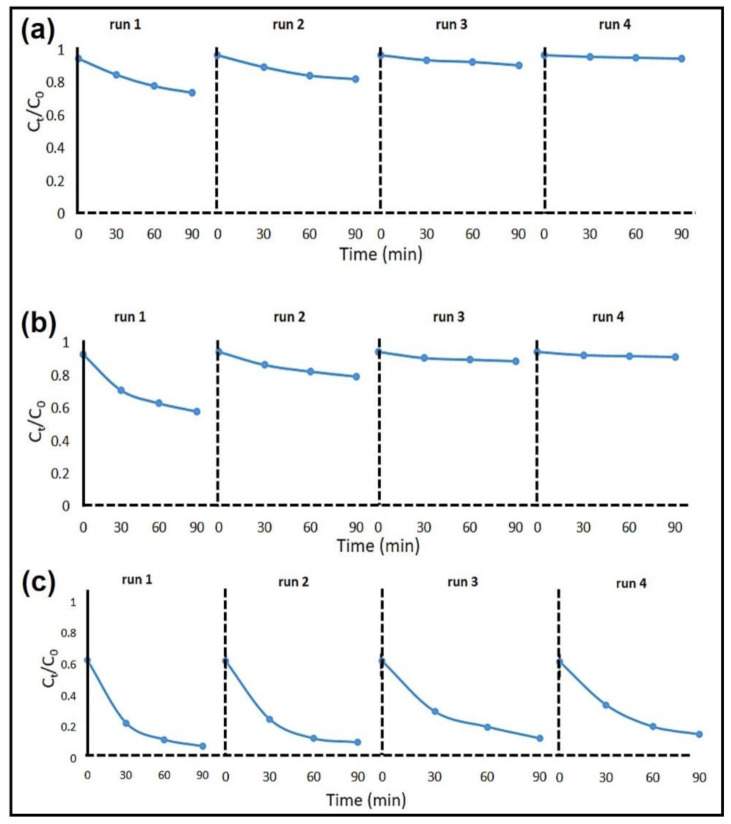
Recycling studies of (**a**) CdS, (**b**) Ag_3_PO_4_, and (**c**) Pt-Ag_3_PO_4_/CdS/chitosan photocatalyst for the photodegradation of MB solution.

Figure 9c shows the MB solution photodegradation in the four successive runs under the same condition. After each run, Pt-Ag_3_PO_4_/CdS/chitosan photocatalyst was washed with double distilled water several times, and a fresh solution of the dye was applied for the next run. The percentage of MB removal of Pt-Ag_3_PO_4_/CdS/chitosan photocatalyst was 92%, 90%, 87%, and 84% after 90 min of illumination time, respectively. It is important to note that the efficiency of photocatalytic activity of the synthesized nanocomposite had only a small reduction after four consecutive tests, indicating that the photocatalyst functioned as an effective catalyst with significant stability. Comparatively, the photodegradation of MB in the presence of pure CdS and Ag_3_PO_4_ for the four types of recycling was about 26%, 19%, 11%, 7% and 42%, 22%, 13%, 10% after 90 min of visible light illumination, respectively (Figure 9a,b).

According to the acquired results, the NPs of CdS and Ag_3_PO_4_ have poor stability against photocorrosion; low structural stability of pure Ag_3_PO_4_ has been extensively explored earlier [79,80,81,82,83], where an excess of metallic Ag^0^ species on the surface of Ag_3_PO_4_, resulting from the photocorrosion, sharply degraded the photochemical stabilities and shielded the light. Figure S4 illustrates the XRD patterns for Pt-Ag_3_PO_4_/CdS/chitosan nanocomposite before and after photodegradation process; a weak peak at 2θ ~38.3° appeared after four cycling runs ascribable to the (111) crystalline plane of metallic Ag (JCPDS No. 65-2871), indicating that a little amount of Ag^0^ species formed on the catalyst’s surface. The ensuing Ag^0^ species, which deposited on the surface of the catalyst, could scatter the photoinduced electrons, inhibited the further photoreduction of Ag^+^ ions, thereby promoting separation of charge carriers [33,83]. The immobilization of CdS and Ag_3_PO_4_ NPs on the chitosan matrix could remarkably inhibit the dissolution of CdS and Ag_3_PO_4_ during the photoreaction assay, thus contributing to the stability of aforementioned photocatalyst. Moreover, the presence of Pt NPs on the surface of catalysts could trap the photogenerated electrons as they improve the photocatalytic property of the Pt-Ag_3_PO_4_/CdS/chitosan nanocomposite [84,85,86,87,88,89,90].

### 3.6. Antibacterial Tests

Cadmium sulfide (CdS) as another well-known narrow bandgap semiconductor has been confirmed to be capable of promoting the carrier transfer and increasing the light absorption [91] when combined with other materials such as AgNO_3_. Thus, the nanocomposite preparation of Ag_3_PO_4_ and CdS was expected to enhance photocatalytic activity [92]. In addition, decorating the prepared photocatalysts with noble metal NPs, such as Pt, is ideal way to increase the photocatalytic activity and photocatalytic antibacterial properties. So, Pt-Ag_3_PO_4_/CdS/chitosan nanocomposite improves the utilization efficiency of light for antibacterial activity under visible light [93]. 

**Figure 10:**
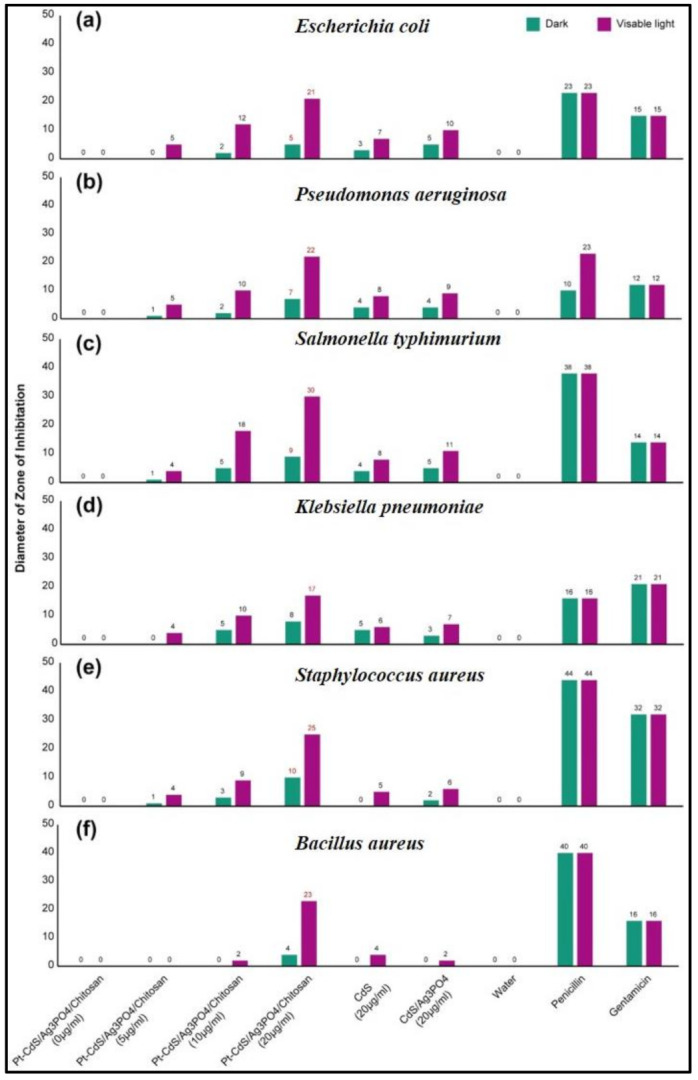
Antibacterial activity study of the prepared samples at different concentrations on the model (**a**) *Escherichia coli*, (**b**) *Pseudomonas aeruginosa*, (**c**) *Salmonella typhimurium*, (**d**) *Klebsiella pneumoniae*, (**e**) *Staphylococcus aureus*, and (**f**) *Bacillus cereus* bacterium under dark and visible light were tested by the well diffusion method based on diameter of zone of inhabitation (mm). Water is negative control.

To investigate the antibacterial activity, the well disk-diffusion method was used to detect the standard antibiotics such as *Penicillin* and *Gentamicin* that have inhibitory action with outstanding zone of inhibition on the microorganisms and used for the examination of *Escherichia coli*, *Pseudomonas aeruginosa*, *Salmonella typhimurium*, *Staphylococcus aureus*, *Klebsiella pneumoniae*, and *Bacillus cereus* bacteria in a Tryptic soy agar medium. In this work, the antibacterial activity of Pt-Ag_3_PO_4_/CdS/chitosan nanocomposite was investigated from 0 to 20 µg/mL under visible light and in the dark; at a concentration of 20 µg/mL, very good results were obtained. The antibacterial activity in the dark was much lower than in the light. The experiments were carried out in contrast with CdS, CdS-Ag_3_PO_4_, standard antibiotic *Penicillin*, and *Gentamicin* using (H_2_O as a negative control) by measuring zone of inhibition in mm and the experimental outcomes are shown in Figure 10. Antibacterial activity by Pt-Ag_3_PO_4_/CdS/chitosan nanocomposite against six bacterial pathogens is due to the release of ROS (reactive oxygen species), which causes damage to the nucleic acids and membrane proteins [65,94,95]. Figure S5 depicts the antibacterial activity of the prepared samples at 20 µg/mL under visible light tested by a well diffusion method on the model of different bacteria.

### 3.7. Cytotoxicity Assays

**Figure 11:**
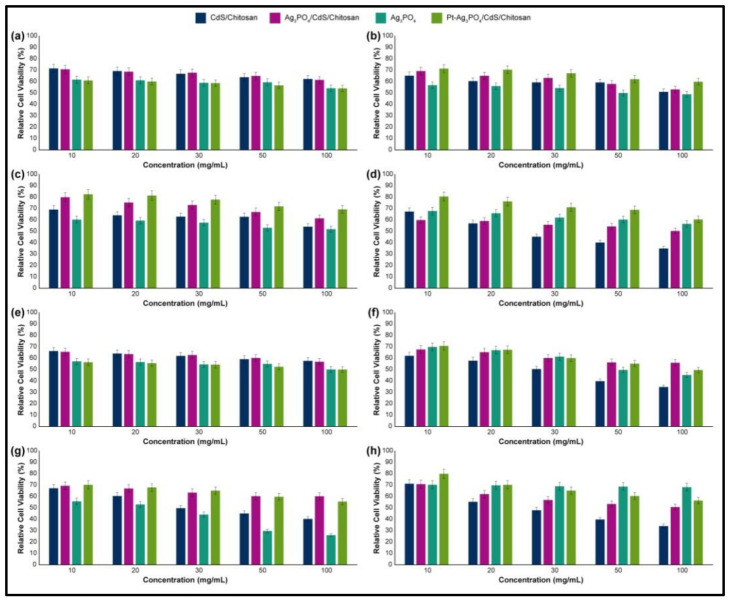
MTT results of the CdS/Chitosan, Ag_3_PO_4_/CdS/chitosan, Ag_3_PO_4_, and Pt-Ag_3_PO_4_/CdS/chitosan on (**a**) HEK-293, (**b**) HeLa, (**c**) HepG2, and (**d**) PC12 cell lines after 24 h of treatment; and on (**e**) HEK-293, (**f**) HeLa, (**g**) HepG2, and (**h**) PC12 after 48 h of treatment.

The cell viability in different cell lines was investigated after treatment (24- and 48-h time points) with the nanocomposite, and for each part of the nanosystem (Figure 11). The results clearly showed that the Ag_3_PO_4_ part has cell viability of more than (61%, 56%, 60%, and 67%) in low concentrations and (54%, 47%, 51%, and 56%) in the highest concentrations (results of HEK-293, HeLa, HepG2, and PC12 cell lines, respectively, after 24 h of treatment), and the cell viability of more than (57%, 69%, 55%, and 70%) in low concentrations and (50%, 45%, 26% and 68%) in the highest concentrations (results of HEK-293, HeLa, HepG2 and PC12 cell lines, respectively, after 48 h of treatment). By addition of this part, Ag_3_PO_4_, to the CdS/chitosan nanocomposite, the cell viability of the CdS/chitosan changes from (71%, 65%, 69%, and 67%) to (70%, 69%, 79%, and 59%) in low concentrations and from (62%, 51%, 54%, and 34%) to (61%, 53%, 61%, and 50%) in the highest concentrations (results of HEK-293, HeLa, HepG2 and PC12 cell lines, respectively, after 24 h of treatment). By addition of this part, Ag_3_PO_4_, to the CdS/chitosan nanocomposite, the cell viability of the CdS/chitosan changes from (66%, 62%, 67%, and 71%) to (65%, 67%, 69%, and 70%) in low concentrations and from (57%, 34%, 40%, and 34%) to (56%, 56%, 60%, and 50%) in the highest concentrations (results of HEK-293, HeLa, HepG2 and PC12 cell lines, respectively, after 48 h of treatment). Interestingly, the role of the addition of the Ag_3_PO_4_ to the carbon-based substrate of CdS/chitosan showed gradual increases in the cell viability, and addition of the Pt to the nanocomposite leads to significant increase in the cell viability. Overall, the cell viability of the final nanocomposite, Pt-Ag_3_PO_4_/CdS/chitosan, was recorded as more than 75% in low concentrations and 61% in high concentrations in both 24 and 48 h of treatment. Based on the available literature [96,97], CdS NPs can endorse the cytotoxic effect in different cell lines including HepG2, HeLa, PC12, and HEK-293 cell lines. However, in this study, the cytotoxicity effect promoted by addition of CdS was much lower, which may be because of the unavailable CdS sites on the surface. Additionally, Ag_3_PO_4_ could also considerably elevate cytotoxicity in the mentioned cell lines [98,99]. In this study, however, some cytotoxicity was discerned because of Ag_3_PO_4_, but it was not in the significant range documented in the literature.

## 4. Conclusions

In summary, the present study describes a facile and inexpensive method to prepare Pt-Ag_3_PO_4_/CdS/chitosan nanocomposite, wherein the synergistic effect among Pt, CdS, Ag_3_PO_4_, and chitosan could increase the response of the composite to visible light-induced photocatalysis and antibacterial activities. These outstanding performances can be related to several factors. Coupling of CdS and Ag_3_PO_4_ could promote the separation of charge carriers. Consequently, the CdS/Ag_3_PO_4_ composite does not suffer from photo corrosion during the photodegradation process. Furthermore, the immobilization of CdS and Ag_3_PO_4_ NPs on the matrix of chitosan could significantly inhibit CdS and Ag_3_PO_4_ from dissolution during the photoreaction, thus promoting the stability of the photocatalyst. Moreover, chitosan can play a significant role in the adsorption of organic pollutants and it is helpful in improving the efficiency of the prepared nanocomposite.

The presence of Pt NPs on the surface of the prepared composite can delay the recombination rate of e^–^/h^+^ pairs. These NPs with large work functions have been used as the electron sinks to trap the photoinduced electrons from the CB of Ag_3_PO_4_, thus prolonging the lifetime of the photoinduced charge carriers. Consequently, the prepared Pt-Ag_3_PO_4_/CdS/chitosan nanocomposite effectively depressed the recombination of e^−^/h^+^ charge carriers and improved the photocatalytic activity, while avoiding the photocorrosion of Ag_3_PO_4_ (Ag^+^ + e^−^ → Ag). The antibacterial experiments exhibit that the the Pt-Ag_3_PO_4_/CdS/chitosan nanocomposite is endowed with considerable antibacterial activity against six types of gram-positive and gram-negative bacteria in a short time under exposure to the visible light, a significant advantage being the low consumption of nanocomposite for attaining the antibacterial activity. The loading of Pt NPs can enhance the antibacterial activity of nanocomposite in the dark and under the visible light. Hence, this work describes an ideal approach to prepare a new and useful nanocomposite of Pt-Ag_3_PO_4_/CdS/chitosan for multiple appliances and may stimulate further innovative combinations.

## Supplementary Materials

The following are available online at https://www.mdpi.com/2079-4991/10/11/2320/s1, Figure S1: The emission wavelength of the light source of NVSWE21AT; Figure S2: FT-IR spectrum of Ag_3_PO_4_; Figure S3: XRD patterns of Ag_3_PO_4_; Figure S4: XRD pattern of Ag_3_PO_4_/CS/CdS nanocomposites before and after four cycling runs; Figure S5: Antibacterial activity picture of the prepared samples at 20 µg/mL under visible light were tested by the well diffusion method on the model of different bacterium (a) *E. coli*, (b) *Pseudomonas* *aeruginosa,* (c) *Salmonella typhimurium,* (d) *Klebsiella pneumonia,* (e) *Staphylococcus aureus,* and (f) *Bacillus cereus*.
