# Supplementary material for: Novel Pt-Ag3PO4/CdS/Chitosan Nanocomposite with Enhanced Photocatalytic and Biological Activities
Source: Nanomaterials (Basel). 2020 Nov 23;10(11):2320. doi: 10.3390/nano10112320 (PMC7700689; doi:10.3390/nano10112320)
Supplement: Supplementary file 1 [file nanomaterials-10-02320-s001.pdf]

Supplementary Materials

Novel Pt-Ag_3PO_4/CdS/Chitosan Nanocomposite with Enhanced Photocatalytic and Biological Activities

Mahsa Kiani [1], Mojtaba Bagherzadeh [1,*], Reyhaneh Kaveh [1], Navid Rabiee [1], Yousef Fatahi [2,3,4], Rassoul Dinarvand [2,3], Ho Won Jang [5], Mohammadreza Shokouhimehr [5,*], and Rajender S. Varma [6,*]

[1] Department of Chemistry, Sharif University of Technology, P.O. Box 11155-3516, Tehran 14155-6451, Iran; mahsa.kiani88@gmail.com (M.K.); reyhanehkaveh@yahoo.com (R.K.); nrabiee94@gmail.com (N.R.)
[2] Department of Pharmaceutical Nanotechnology, Faculty of Pharmacy, Tehran University of Medical Sciences, Tehran 14155-6451, Iran; youseffatahi@gmail.com (Y.F.); dinarvand@tums.ac.ir (R.D.)
[3] Nanotechnology Research Center, Faculty of Pharmacy, Tehran University of Medical Sciences, Tehran 14155-6451, Iran
[4] Universal Scientific Education and Research Network (USERN), Tehran 15875-4413, Iran
[5] Department of Materials Science and Engineering, Research Institute of Advanced Materials, Seoul National University, Seoul 08826, Korea; hwjang@snu.ac.kr
[6] Regional Center of Advanced Technologies and Materials, Palacky University, Šlechtitelů 27, 78371 Olomouc, Czech Republic
* Correspondence: bagherzadeh@sharif.edu (M.B.); mrsh2@snu.ac.kr (M.S.); varma.rajender@epa.gov (R.S.V.)

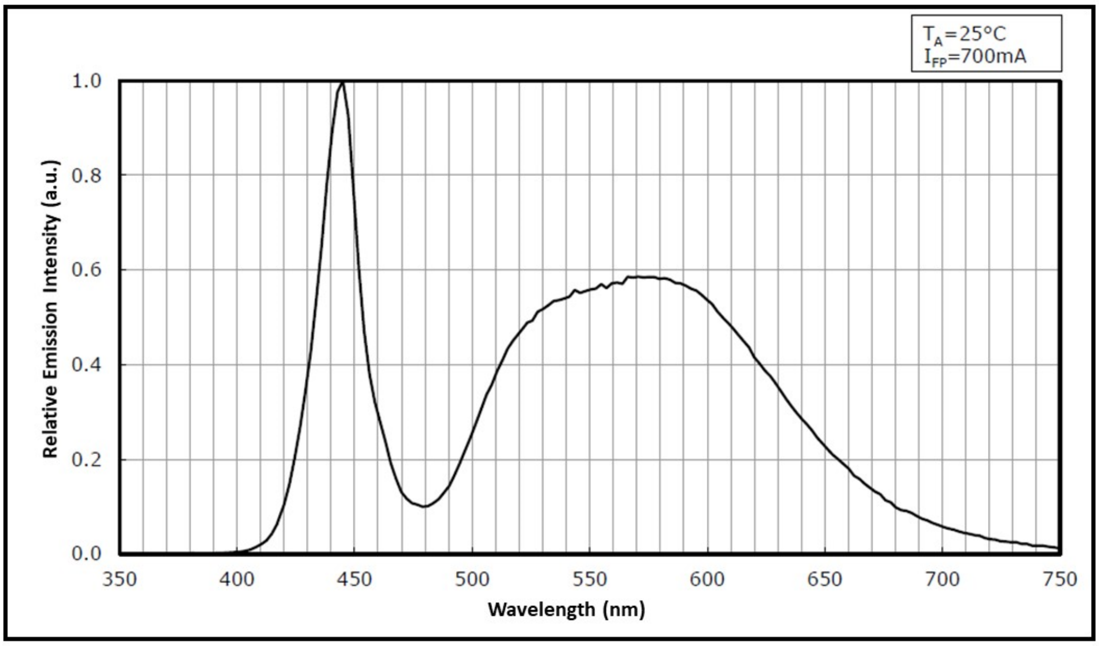

Figure S1. The emission wavelength of the light source of NVSWE21AT.

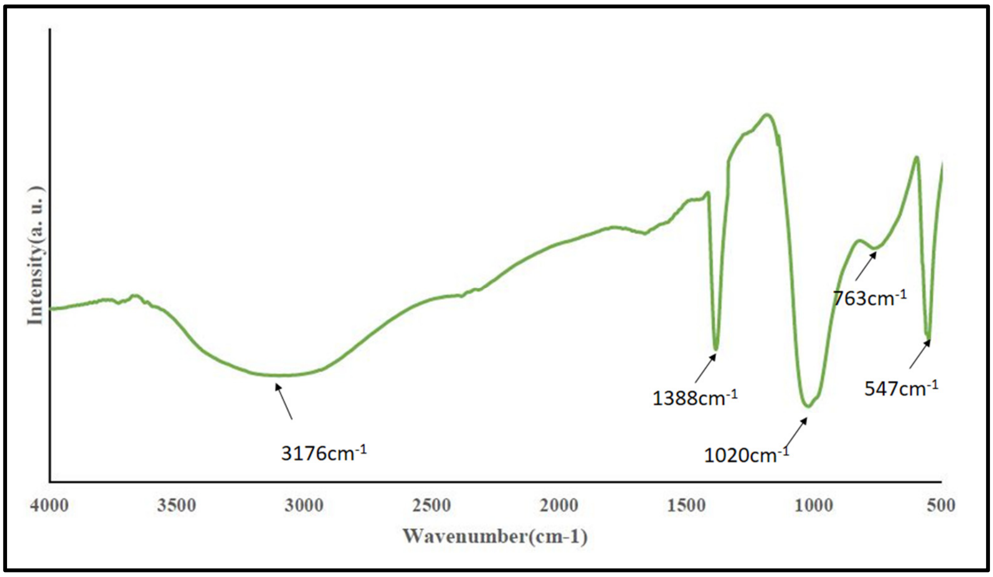

Figure S2. FT-IR spectrum of Ag_3PO_4 [1].

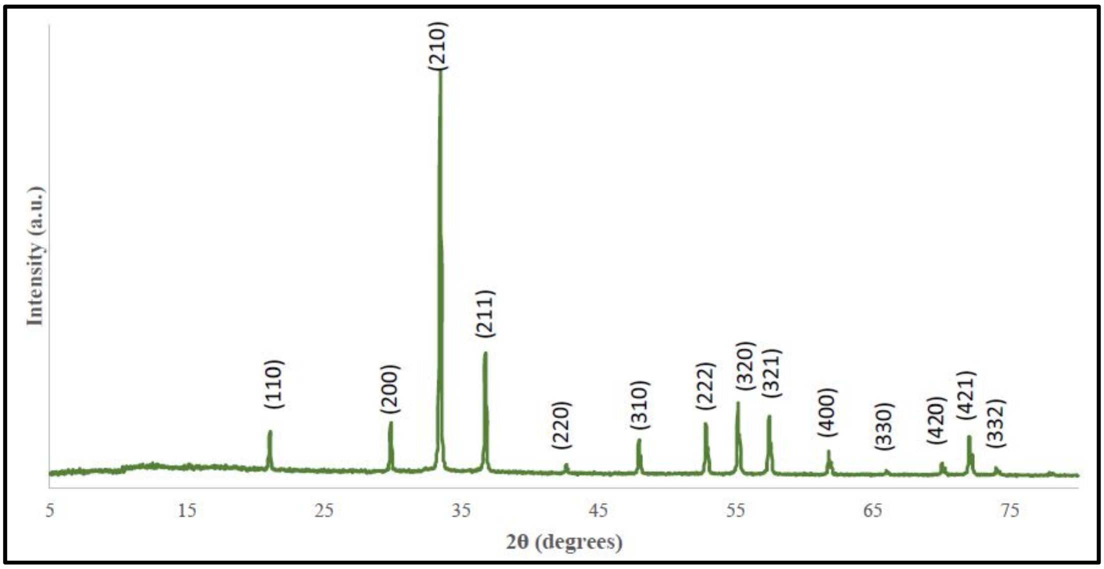

Figure S3. XRD patterns of Ag_3PO_4. Reprinted with permission from[1].

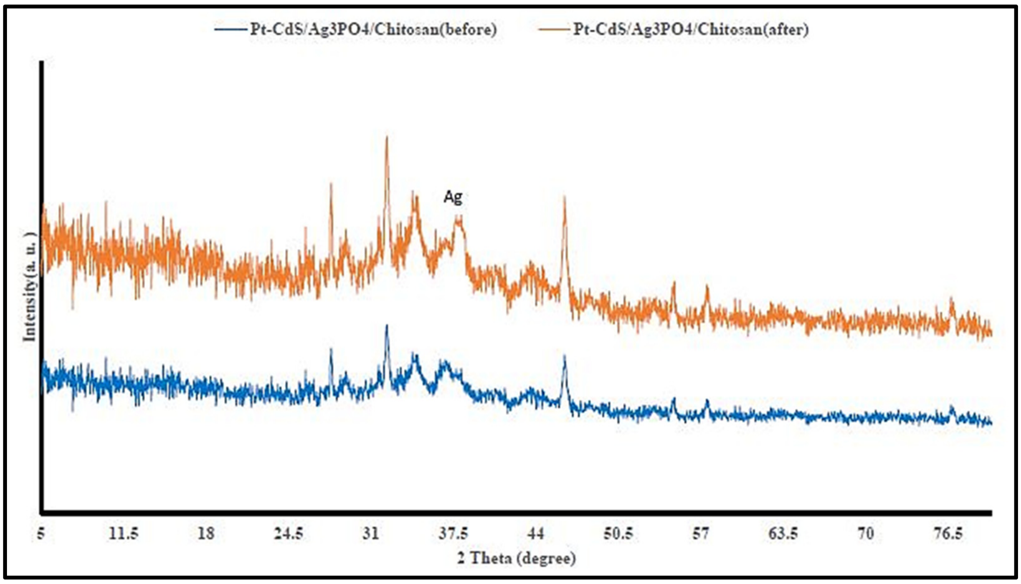

Figure S4. XRD pattern of Ag_3PO_4/CS/CdS nanocomposites before and after four cycling runs.

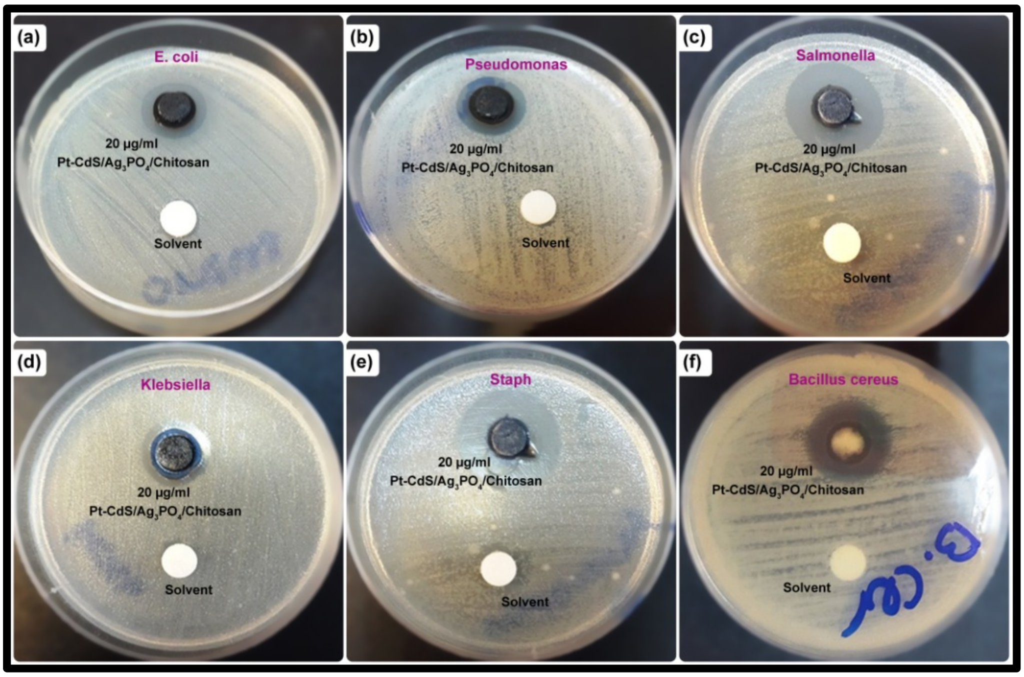

Figure S5. Antibacterial activity picture of the prepared samples at 20 µg/mL under visible light were tested by well diffusion method on the model of assorted bacterium (**a**) *E. Coli* (**b**) *Pseudomonas aeruginosa* (**c**) *Salmonella typhimurium* (**d**) *Klebsiella pneumonia* (**e**) *Staphylococcus aureus* (**f**) *Bacillus cereus*.

References:

1. Bagherzadeh, M.; Kaveh, R. New Magnetically Recyclable Reduced Graphene Oxide rGO/MFe_2O_4 (M= Ca, Mg)/Ag_3PO_4 Nanocomposites With Remarkably Enhanced Visible-light Photocatalytic Activity and Stability. *Photochem. Photobiol.* **2018**, *94*, 1210–1224.
